# Integrated Volatile Phytochemical Profiling of Wild *Helichrysum italicum* subsp. *italicum* Populations from the Tuscan Archipelago

**DOI:** 10.3390/molecules31193577

**Published:** 2026-10-08

**Authors:** Weronika Adler, Michele Moretta, Enrico Palchetti, Giulia Vanti, Stefania Nin, Anna Rita Bilia

**Affiliations:** 1 Department of Chemistry “Ugo Schiff”, University of Florence, 50019 Sesto Fiorentino, Italy; weronika.adler@unifi.it (W.A.); giulia.vanti@unifi.it (G.V.); ar.bilia@unifi.it (A.R.B.); 2 Department of Agriculture, Food, Environment and Forestry, University of Florence, Piazzale delle Cascine, 18, 50144 Firenze, Italy; enrico.palchetti@unifi.it; 3 Research Centre for Vegetable and Ornamental Crops, Council for Agricultural Research and Economics (CREA), Via dei Fiori 8, 51017 Pescia, Italy; stefania.nin@crea.gov.it

**Keywords:** *Helichrysum italicum*, essential oil, GC–MS, HS-SPME–GC–MS, volatile profile, Tuscan Archipelago, hydrosol

## Abstract

*Helichrysum italicum* (Roth) G. Don subsp. *italicum* is one of the most valuable Mediterranean aromatic species owing to its essential oil, which is widely used in pharmaceutical, cosmetic, and nutraceutical applications. However, comparative phytochemical investigations simultaneously addressing essential oils and hydrosols from geographically isolated natural populations remain limited. In this study, we investigated essential oils and hydrosols from pooled plant material collected at six sites across the Tuscan Archipelago (Capraia, Elba, and Giglio Islands) by gas chromatography–mass spectrometry (GC–MS) and headspace solid-phase microextraction coupled with GC–MS (HS-SPME–GC–MS). The analyzed pooled site samples shared an essential-oil profile dominated by neryl acetate (24.90–34.01%), γ-curcumene (9.10–14.91%), α-pinene (5.05–14.17%), and neryl propionate (4.37–9.14%), while their relative abundances differed among the analyzed pooled site samples. Marked differences were observed among the hydrosol headspace profiles. Within the HS-SPME–GC–MS profiles, eucalyptol showed the highest relative peak-area contribution in the four Capraia pooled samples (39.13–94.56%), whereas the Elba profile was dominated by neryl acetate (56.36%). The Giglio profile was mainly characterized by nerol (29.81%), fenchyl alcohol (21.72%), and 4-terpineol (16.56%). Overall, the results provide complementary descriptive information on the volatile composition of the analyzed pooled site samples and identify candidate compositional patterns requiring validation through independent biological sampling and replicated distillations.

## 1. Introduction

The genus *Helichrysum* Mill. comprises more than 600 species distributed mainly across the Mediterranean region, Europe, Africa, and parts of Central and Western Asia [1]. Among Mediterranean taxa, *Helichrysum italicum* (Roth) G. Don is one of the most extensively investigated species because of its ecological adaptability, aromatic properties, and growing economic relevance. Commonly known as immortelle, *H. italicum* is a drought-tolerant perennial shrub that occurs naturally in arid, sandy, and rocky habitats typical of Mediterranean ecosystems. The species is particularly valued for its essential oil and specialized metabolites, which are widely employed in cosmetic, pharmaceutical, nutraceutical, and phytotherapeutic applications. Several studies have reported antioxidant, antimicrobial, anti-inflammatory, and antiproliferative activities from extracts and essential oils of *H. italicum* [2,3,4,5,6]. This growing scientific and commercial interest has increased attention to phytochemical variability, traceability, and the sustainable exploitation of natural populations.

Despite its ecological and economic importance, the taxonomy of *Helichrysum* remains challenging because of the extensive morphological plasticity, overlapping diagnostic characters, and frequent natural hybridization among Mediterranean taxa [7,8,9]. Traditional classifications based exclusively on morphological traits have therefore undergone several revisions as molecular and phylogeographic evidence has been integrated. Recent studies show that genetic diversity within the *H. italicum* complex is geographically structured, prompting a re-evaluation of subspecies delimitation and distribution across the Mediterranean Basin [8,10]. In this context, phytochemical characterization has become an important complementary approach to classical taxonomy, providing valuable chemotaxonomic markers that can support species discrimination, population characterization, and the interpretation of evolutionary relationships [11].

The Tuscan Archipelago represents an important center of plant biodiversity in the Mediterranean Basin, where geographical isolation, heterogeneous environmental conditions, and limited gene flow have promoted the differentiation of numerous endemic and native plant populations. Within this archipelago, *Helichrysum italicum* subsp. *italicum* is naturally distributed on several islands, including Capraia, Elba and Giglio, where populations occupy habitats differing in altitude, substrate, exposure and climatic conditions [12,13,14]. Recent floristic, morphological, and micromorphological investigations confirmed that populations on these islands belong to *H. italicum* subsp. *italicum*, while highlighting appreciable variability among geographically isolated populations [13,14]. These insular populations provide suitable material for exploratory, descriptive characterization of phytochemical profiles across different sampling sites. However, assessing the effects of geographical isolation or local environmental conditions would require replicated environmental measurements and independent biological sampling.

The phytochemical characterization of *H. italicum* has traditionally focused on essential oils because of their commercial value and biological properties. Hydrodistillation also produces a condensed aqueous phase known as a hydrosol, hydrolate, aromatic water, or floral water. Hydrosols may contain small quantities of dissolved essential-oil constituents, relatively water-soluble volatile compounds, and traces of other co-distilled plant components.

Hydrosols have recently attracted increasing interest owing to their biological activities and potential applications in cosmetics, food preservation, and phytotherapy [15]. Nevertheless, their volatile composition remains considerably less investigated than that of essential oils, particularly in wild Mediterranean populations. Joint characterization of essential oils and hydrosol volatile fractions can therefore provide complementary information on the phytochemical diversity of natural populations.

Although previous studies have investigated the morphology, taxonomy, and essential-oil composition of *Helichrysum italicum* populations from the Tuscan Archipelago, direct comparisons between essential oils and hydrosol volatile fractions from geographically isolated natural populations remain limited. The present study therefore aimed to characterize six wild populations of *H. italicum* subsp. *italicum* collected on Capraia, Elba, and Giglio Islands. We analyzed essential oils by GC–MS, and investigated hydrosol volatile fractions by HS-SPME–GC–MS. The resulting profiles were descriptively examined to identify candidate site-associated compositional patterns. Given the exploratory sampling design, the study was not intended to establish causal relationships between chemical composition and geographical, environmental, or genetic factors.

## 2. Results and Discussion

### 2.1. Hydrodistillation of Helichrysum italicum Populations

Hydrodistillation of 1606–2183 g of fresh aerial biomass produced 2.20–4.00 mL of essential oil (EO), corresponding to apparent yields of 0.14–0.19% (*v*/*w*, fresh weight) (Table 1). The highest nominal yields were recorded for CIV and E (0.19%), followed by CIII (0.17%), G (0.16%), and CI and CII (0.14%). Because each value was derived from a single bulk sample and no replicate distillations or measures of dispersion were available, these differences are descriptive and should not be interpreted as statistically supported population effects. Comparisons with published yields also require caution because the plant organs included, phenological stage, moisture status, storage conditions, and distillation configuration can all affect EO recovery [16].

### 2.2. Characterization of the Essential Oils by Gas Chromatography–Mass Spectrometry (GC–MS)

**Figure 1:**
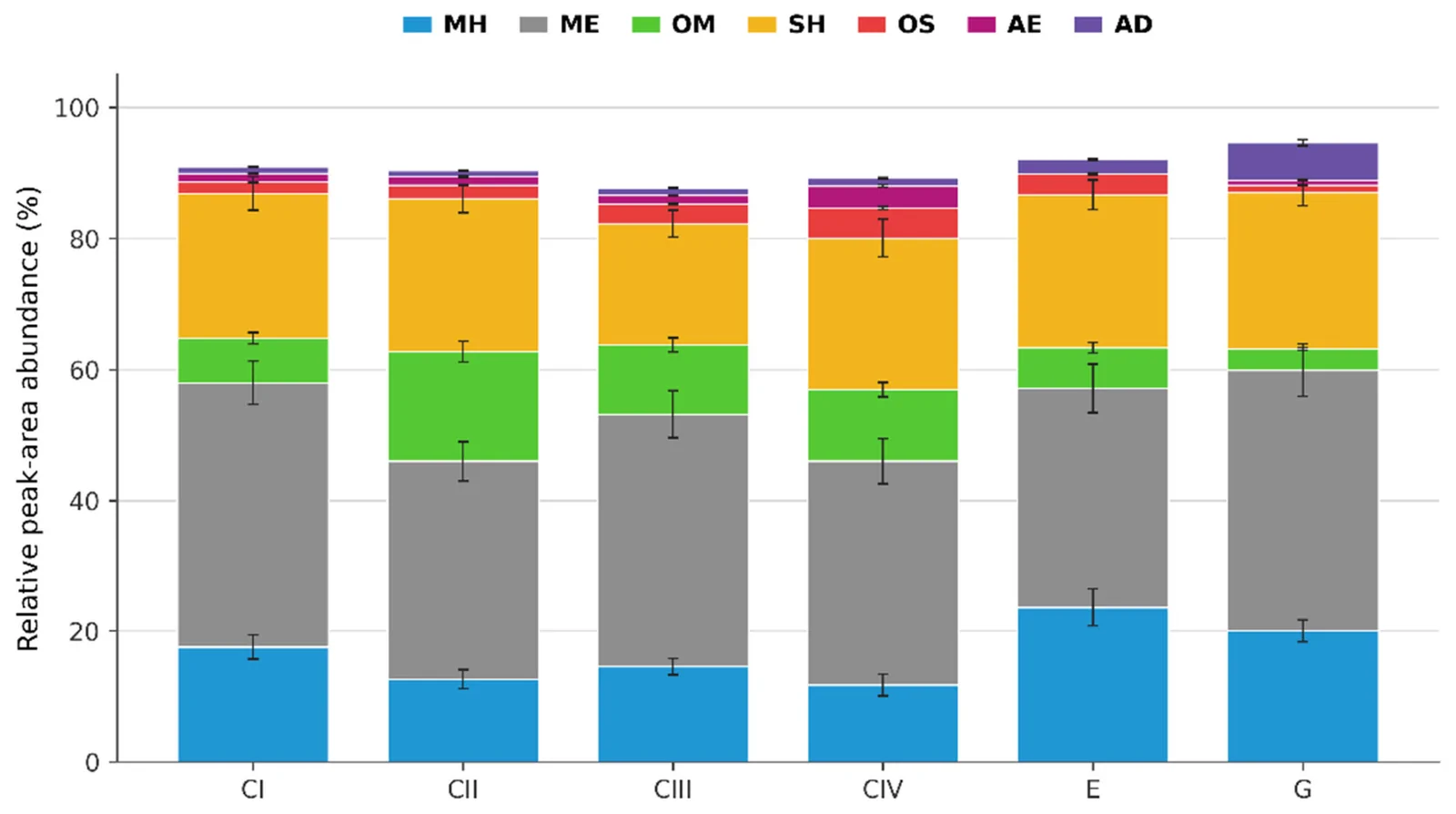
Distribution of the main chemical classes identified in the essential oils obtained from the six pooled site samples. Values represent mean relative peak-area abundances, and error bars indicate the standard deviation of three technical GC–MS replicates. CI–CIV, Capraia samples; E, Elba; G, Giglio. MH, monoterpene hydrocarbons; ME, monoterpene esters; OM, oxygenated monoterpenes; SH, sesquiterpene hydrocarbons; OS, oxygenated sesquiterpenes; AE, aliphatic esters; AD, aliphatic diketones. Technical replicates assess analytical repeatability and do not represent biological replication.

GC–MS analyses identified 19 to 25 components of the essential oil samples (Table 2). Monoterpenes accounted for 56.96–64.77% of the identified fraction, whereas sesquiterpenes represented 21.49–26.55% (Table 2). A detailed picture of the different classes of constituents, namely MH, monoterpene hydrocarbon; ME, monoterpene ester; OM, oxygenated monoterpene; SH, sesquiterpene hydrocarbon; OS, oxygenated sesquiterpene; AE, aliphatic ester; and AD, aliphatic diketone (Figure 1). Despite this broad similarity in compound-class distribution, the relative abundance of individual constituents varied among populations. Four constituents—neryl acetate, γ-curcumene, α-pinene, and neryl propionate—were detected in every EO and together accounted for 51.04–62.18% of the identified volatile fraction. Neryl acetate was the most abundant compound in all samples (24.90–34.01%), followed by γ-curcumene (9.10–14.91%). α-Pinene varied from 5.05 to 14.17%, while neryl propionate ranged from 4.37 to 9.14%. The recurrence of neryl acetate and γ-curcumene among the dominant constituents is consistent with previously reported data for *H. italicum* subsp. *italicum* from the Tuscan Archipelago and other Mediterranean areas [17,18,19,20,21,22,23]. These studies document substantial variation in neryl derivatives, α-pinene, γ-curcumene, and other mono- and sesquiterpenes across plant material, geographic origins, taxa, and analytical approaches.

The clearest quantitative contrasts concerned G and E. The Giglio sample contained the highest proportions of neryl acetate (34.01%), γ-curcumene (14.91%), and limonene (9.71%), whereas the Elba sample had the highest α-pinene content (14.17%). Variation was also observed among the four Capraia samples: CII had the highest eucalyptol (7.98%) and linalool (5.03%) contents, while CIV combined the lowest α-pinene value (5.05%) with the highest rosifoliol value (4.55%). These descriptive differences indicate that the analyzed pooled samples shared a common set of dominant compounds, although their relative abundances varied among samples.

Several low-abundance compounds showed an apparently restricted occurrence. β-Farnesene and p-cymene were detected only in CI, longipinene only in CII, and ylangene and 3,5,7,10-tetramethyl-9-undecene-4,6-dione only in G. Given their low relative abundance, tentative library-based identification, and the absence of replicate analyses, these compounds should be considered candidate discriminatory features rather than validated chemotaxonomic markers.

Overall, the analyzed EO samples shared a neryl-acetate/γ-curcumene-rich profile, with descriptive differences in the relative abundance of individual compounds rather than clearly separated chemotypes. A recent survey of 316 individuals from 12 Tuscan Archipelago populations likewise reported substantial variation in inflorescence terpene composition, with a greater proportion of variation occurring among populations than within populations [19]. Comparable associations among chemical profiles, population origin, and genetic structure have been reported in Italian and Sardinian germplasm [24,25]. The present analysis, however, was based on one pooled distillation per population; it therefore cannot partition within-population variability or distinguish genetic effects from environmental, phenological, and processing effects. Replicated sampling and common-garden evaluation would be required before population-specific profiles could be used for germplasm selection or product authentication.

### 2.3. Characterization of the Hydrosols by Headspace Solid-Phase Microextraction Coupled with Gas Chromatography–Mass Spectrometry (HS-SPME–GC–MS)

HS-SPME–GC–MS profiles were obtained for hydrosols from all six populations (CI–CIV, E, and G), as reported in Table 3. Across the complete dataset, 24 volatile compounds were identified, while compound richness ranged from five constituents in CI and E to 17 in CIV. The six hydrosol headspace profiles showed marked differences in the number of detected compounds and their relative peak-area distributions under the same HS-SPME–GC–MS conditions.

The four pooled hydrosol samples from Capraia showed distinct relative peak-area profiles, particularly in the contribution of eucalyptol and other oxygenated volatiles. CI had the simplest profile, with five detected compounds, and eucalyptol accounted for 94.56% of the total identified HS-SPME–GC–MS peak area. CII was likewise dominated by eucalyptol (87.01%), with minor amounts of neryl acetate (4.23%), linalool (2.85%), 4-terpineol (2.45%), fenchyl alcohol (2.15%), and nerol (1.31%). In CIII, eucalyptol decreased to 46.94%, while neryl acetate (17.32%), nerol (8.79%), geranyl propionate (8.01%), linalool (6.95%), α-curcumene (4.78%), fenchyl alcohol (4.21%), and 4-terpineol (3.00%) contributed more.

CIV displayed the greatest compound richness, with 17 detected constituents. Eucalyptol remained the largest peak (39.13%), followed by neryl acetate (18.99%), linalool (9.60%), geranyl propionate (6.30%), α-curcumene (5.93%), nerol (4.79%), and fenchyl alcohol (4.30%); the aliphatic diketone 2,4-dimethylheptane-3,5-dione (0.43%) was detected only in CIV. The E hydrosol was instead dominated by neryl acetate (56.36%), α-curcumene (20.59%), and geranyl propionate (11.50%). The G hydrosol was rich in oxygenated monoterpenes, particularly nerol (29.81%), fenchyl alcohol (21.72%), 4-terpineol (16.56%), and linalool (11.31%), with smaller contributions from rosifoliol (6.23%), bisabolane (4.41%), endo-borneol (1.98%), guaiol (1.07%), and α-eudesmol (0.79%).

The EO and hydrosol datasets were obtained using different sample-introduction procedures, and their relative peak-area percentages cannot therefore be compared quantitatively. Nevertheless, the hydrosol headspace profiles showed a high relative contribution of oxygenated monoterpenes. Within the HS-SPME–GC–MS dataset, eucalyptol accounted for 94.56%, 87.01%, 46.94%, and 39.13% of the total identified peak area in CI, CII, CIII, and CIV, respectively. These values reflect compound abundance within each chromatographic profile and do not represent absolute concentrations in the liquid hydrosols. Previous studies have similarly shown that the number and relative abundance of compounds detected in *H. italicum* hydrosols are strongly dependent on the analytical procedure adopted [1,26]. These percentages should not be interpreted as absolute aqueous concentrations, because HS-SPME responses depend on analyte partitioning, fiber affinity, extraction conditions, and matrix effects.

Thus, the six hydrosol profiles provided discriminatory information complementary to that obtained from the EO analysis. Nevertheless, the present sampling design does not allow the observed compositional patterns to be attributed to geographical isolation, local environmental conditions, genetic differentiation, individual variability, or distillation-related effects. The profiles identify candidate compositional differences for future targeted validation; sensory properties, biological activity, and commercial quality should be assessed experimentally rather than inferred from relative composition alone.

### 2.4. Radical Scavenging Assay by DPPH^•^

The antioxidant activity of the EO and hydrosol samples was evaluated using the DPPH radical-scavenging assay, with ascorbic acid as the reference antioxidant. The results, expressed as µg of ascorbic acid equivalents per milliliter (µg AAE mL^−1^), are reported in Table 4. The EO samples showed antioxidant activities ranging from 289.99 ± 70.00 µg AAE mL^−1^ for G to 484.87 ± 67.45 µg AAE mL^−1^ for CIV. Among the Capraia samples, CIV and CIII recorded the highest values, followed by CII and CI. The E and G samples exhibited comparatively lower values. The hydrosols showed substantially lower DPPH radical-scavenging activity, ranging from 1.77 ± 0.32 µg AAE mL^−1^ for G to 4.01 ± 0.58 µg AAE mL^−1^ for CII. Based on the corresponding EO and hydrosol values, EO antioxidant activity was approximately 105.7- to 180.9-fold higher than that of the respective hydrosols. These results are consistent with the order of magnitude reported by Bezek et al. [15], who measured antioxidant activities of 684.66 ± 94.22 µg AAE mL^−1^ for *H. italicum* EO and 4.88 ± 0.59 µg AAE mL^−1^ for the corresponding hydrosol using the DPPH assay. In that study, the EO exhibited more than 100-fold higher antioxidant activity than the hydrosol. Nevertheless, the authors considered the hydrosol’s antioxidant potential appreciable despite its markedly lower activity compared with the concentrated EO.

The lower antioxidant activity of the hydrosols may reasonably reflect the much lower overall abundance of volatile and semi-volatile constituents in the aqueous distillate compared with the concentrated EO. However, the present results cannot establish the contribution of individual compounds. The EO profiles were mainly characterized by neryl acetate and γ-curcumene, whereas the hydrosol headspace profiles differed among the analyzed samples: eucalyptol predominated in CI and CII; eucalyptol and neryl acetate were both represented in CIII and CIV; neryl acetate and α-curcumene characterized E; and oxygenated monoterpenes, particularly nerol, fenchyl alcohol and 4-terpineol, characterized G. These compositional observations should be interpreted cautiously. The relative peak-area percentages obtained by GC–MS and HS-SPME–GC–MS do not represent absolute concentrations and therefore cannot be directly correlated with the DPPH results. Moreover, antioxidant activity may arise from the combined or synergistic effects of multiple constituents, including compounds present at low concentrations. Compound-specific assays and absolute quantification would be required to determine the contribution of individual constituents to the observed radical-scavenging activity.

### 2.5. Agronomic Implications, Germplasm Value, and Study Limitations

The six EOs shared a common set of dominant constituents, but their relative proportions differed among population samples; the six corresponding hydrosols also showed marked descriptive differences in their HS-SPME–GC–MS profiles. These observations identify potentially useful chemical diversity in wild *H. italicum* subsp. *italicum* germplasm from the Tuscan Archipelago. However, the field design confounds population origin with local environment, and each EO and hydrosol profile derives from a single pooled site sample and a single distillation batch, without independent biological or distillation replication. Consequently, altitude, soil, exposure, climate, genetic background, and processing cannot be separated as causal drivers in the present dataset.

Independent regional evidence provides additional context for interpreting these descriptive patterns. Marini et al. [14] reported relatively low genetic differentiation among *Helichrysum* populations from the Tuscan Archipelago, with approximately 9% of the genetic variation occurring among populations and most variation occurring within populations. The authors also suggested that gene flow may limit differentiation among islands. These findings indicate that geographical separation does not automatically correspond to strong genetic differentiation. However, the individuals Marini et al. examined were not directly matched to the pooled plant material analyzed in the present study. Their genetic results therefore cannot be formally correlated with our phytochemical profiles and are cited only as regional contextual evidence.

The observed pattern is nevertheless consistent with broader evidence that *H. italicum* terpene composition varies among Mediterranean populations. In the Tuscan Archipelago, Marini et al. [19] found that variation in inflorescence terpenes was greater among populations than within them. Under common cultivation conditions, Ninčević Runjić et al. [27] also detected persistent population differences in several major EO constituents, supporting a contribution of genetic differentiation and local adaptation in eastern Adriatic germplasm. These studies provide broader contextual evidence of chemical and genetic variability in *H. italicum*, but they do not demonstrate that the differences observed among the pooled samples examined here were caused by genetic differentiation or local environmental conditions. From an applied perspective, the current profiles can guide the selection of candidate populations for a replicated evaluation program, but they are not yet sufficient to define elite germplasm or population-specific quality standards. The sensory and industrial relevance of individual volatiles also depends on odor activity rather than relative abundance alone, as shown by GC-olfactometry of commercial *H. italicum* oil [28]. Evidence from cultivated lavender and lavandin in Tuscany likewise shows that cultivar, site, and year can jointly affect biomass production, essential-oil yield, and chemical composition, underscoring the importance of evaluating aromatic-plant germplasm across environments and growing seasons [29]. A robust selection strategy should compare multiple individuals per population under common environmental conditions, repeat sampling and distillations across years or harvest stages, and combine yield, target-compound abundance, multivariate chemical profiles, and relevant biological or sensory assays. Conserving and testing multiple populations would retain a wider range of biochemical diversity while supporting domestication, traceability, and the development of locally adapted production chains without increasing pressure on wild stands.

## 3. Materials and Methods

### 3.1. Collection of the Plant Material

**Figure 2:**
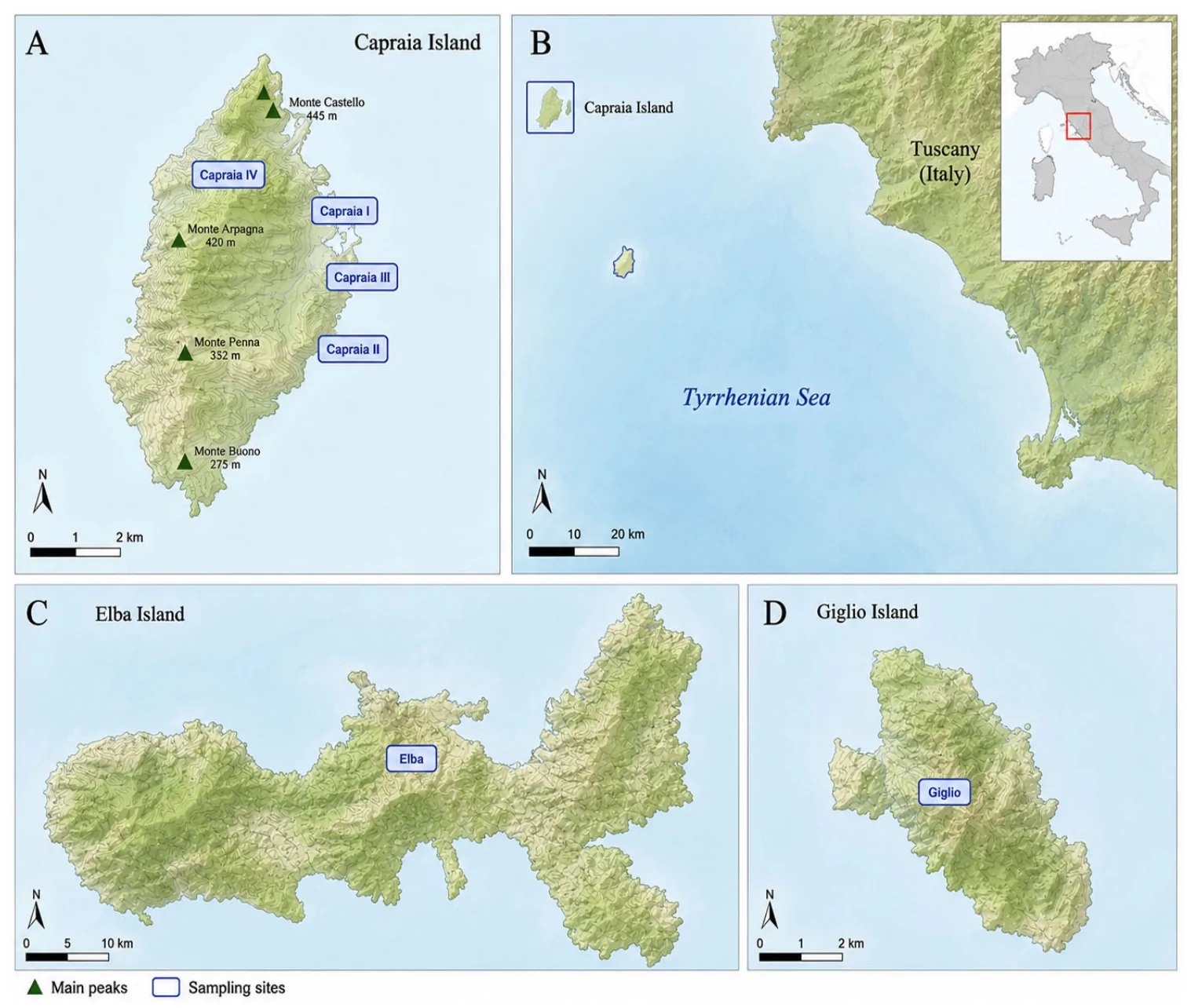
Sampling locations of *Helichrysum italicum* (Roth) G. Don subsp. *italicum* in the Tuscan Archipelago (Central Italy). (**A**) Distribution of the four populations sampled on Capraia Island (CI–CIV). (**B**) Geographic location of the study area within the Tuscan Archipelago, showing the islands of Capraia, Elba, and Giglio. (**C**) Sampling site on Elba Island (E). (**D**) Sampling site on Giglio Island (G). Sampling site codes correspond to those used throughout the manuscript.

Fresh aerial parts (flowering tops and upper leaves) of *Helichrysum italicum* (Roth) G. Don subsp. *italicum* were collected in June 2025 from six wild populations distributed across the Tuscan Archipelago (Capraia, Elba, and Giglio Islands, Central Italy). We harvested approximately 2 kg of fresh aerial biomass from multiple individuals in each population for hydrodistillation. The sampling sites corresponded to locations previously investigated in floristic and phytochemical studies; however, all plant material analyzed in this work came from a new, independent field collection. Sampling followed the protocol described by Marini et al. [13]. We selected sites along a coastal-to-submontane altitudinal gradient to capture the archipelago’s ecological variability. At each locality, we randomly selected flowering individuals within homogeneous stands and spaced them at least 5 m apart to minimize the likelihood of sampling clonally related plants. We collected only individuals at the full flowering stage. Sampling locations, geographic coordinates (WGS84), and altitude are reported in Figure 2 and Table 5. We transported fresh plant material to the laboratory in ventilated containers and processed it within 24 h. Prior to hydrodistillation, the biomass was stored at 4 °C. Voucher specimens were prepared for each population and deposited in the Herbarium of the University of Florence (FI).

### 3.2. Hydrodistillation

EOs were then obtained by hydrodistillation for approximately 90 min using a commercial stainless-steel essential-oil distiller (Spring 12 L, Albrigi Luigi S.r.l., Grezzana, Italy) equipped with a 1600 W induction plate (HA-INDUC-11, König Electronic, ’s-Hertogenbosch, The Netherlands). For each batch, we placed 3 L of water in the base vessel. The harvested stems were cut and arranged evenly on a perforated plate to avoid preferential steam flow, and a second plate was placed on top to keep the biomass compact. Distillation lasted for 90 min, with temperature and essential-oil yield monitored periodically. Approximately 25 mL of aqueous distillate (hydrosol) was recovered per batch. However, because hydrodistillation generates a continuous aqueous distillate phase, hydrolate volume mainly reflected steam condensation and water recovery rather than extraction efficiency; therefore, we considered only EO yield for process evaluation. The obtained oil was dried over anhydrous sodium sulfate and stored in transparent glass vials under refrigeration to prevent degradation and volatilization.

### 3.3. Characterization of Essential Oils by Gas Chromatography–Mass Spectrometry (GC–MS)

GC–MS analyses were performed using an Agilent 7820A gas chromatograph coupled to an Agilent 5977B single quadrupole mass spectrometer (Agilent Technologies, Cernusco sul Naviglio, Milan, Italy) equipped with an electron ionization (EI) source operating at 70 eV. Chromatographic separation was achieved on a DB-5MS capillary column (30 m × 0.25 mm i.d., 0.10 μm film thickness; J&W Scientific, Agilent Technologies) using helium as the carrier gas at a constant flow rate of 1.0 mL min^−1^. Samples (1 μL) were injected in split mode (split ratio 15:1). The injector and transfer line temperatures were maintained at 275 and 280 °C, respectively, while the ion source and quadrupole temperatures were set at 230 and 150 °C. Mass spectra were acquired in full-scan mode over an *m*/*z* range of 29–550 at a scan rate of 2.8 scans s^−1^. The instrument was calibrated according to the manufacturer’s recommendations prior to analysis. Data acquisition and processing were carried out using Agilent MassHunter software (version B.07 SP2; Agilent Technologies). Tentative compound identification was based on comparison of retention times and mass spectra with the Wiley 7 and NIST 20 mass spectral libraries, accepting library matches with a similarity score ≥ 80%. Each pooled essential-oil sample was analyzed in three independently prepared aliquots by GC–MS. Relative peak-area abundances are reported as mean ± standard deviation of the three technical replicates. These technical replicates assess analytical repeatability and do not represent independent biological or distillation replicates.

### 3.4. Characterization of Hydrosols by Headspace Solid-Phase Microextraction Coupled with Gas Chromatography–Mass Spectrometry (HS-SPME–GC–MS)

Hydrosols from all six populations (CI–CIV, E, and G) were analyzed by headspace solid-phase microextraction coupled with gas chromatography–mass spectrometry (HS-SPME–GC–MS) using a 100 μm polydimethylsiloxane (PDMS) fiber (Supelco, Bellefonte, PA, USA). Liquid samples (5 mL) were placed in 20 mL headspace vials and equilibrated at 50 °C for 15 min; the SPME fiber was then exposed to the headspace for 10 min at the same temperature. Thermal desorption was carried out in the GC injector at 270 °C in splitless mode. Compound identification was based on comparison of retention times and mass spectra with reference databases, as reported for the essential-oil analysis. The relative abundance of each compound was expressed as a percentage of the total identified HS-SPME–GC–MS peak area. Three independent HS-SPME extractions were performed for each pooled hydrosol sample under the same experimental conditions. Relative peak-area abundances are reported as mean ± standard deviation of the three technical replicates. These technical replicates assess analytical repeatability and do not represent independent biological or distillation replicates.

### 3.5. DPPH Radical Scavenging Assay

The radical-scavenging capacity of the essential oil (EO) and hydrosol samples was evaluated using the 1,1-diphenyl-2-picrylhydrazyl radical (DPPH^•^) assay, following Bezek et al. [15] with minor modifications. Briefly, 50 µL of hydrosol or 1 mL of a 0.2% (*v*/*v*) EO solution in methanol was mixed with 1 mL of a 0.1 mM DPPH^•^ solution in methanol. The final volume was adjusted to 4 mL with methanol. The mixtures were thoroughly mixed and incubated in the dark at 25 °C for 1 h. Absorbance was subsequently measured at 517 nm using an Agilent 8453 UV–visible diode-array spectrophotometer (Agilent Technologies, Santa Clara, CA, USA). The radical-scavenging activity (RSA) was calculated according to Equation (1):

(1) $$RSA\;(\%)=\frac{A_{0}-A_{s}}{A_{0}}\times 100$$

where $A_{0}$ is the absorbance of the control containing all reagents except the tested sample, and $A_{s}$ is the absorbance measured in the presence of the tested sample. Ascorbic acid was used as the reference antioxidant. Radical-scavenging capacity was calculated using an ascorbic acid calibration curve and expressed as micrograms of ascorbic acid equivalents per milliliter of the original sample (µg AAE mL^−1^). Two independent experiments were performed, each with two technical replicates. Results are reported as mean ± standard deviation of the four measurements. 

## 4. Conclusions

Samples obtained from pooled plant material collected at six sites across the Tuscan Archipelago shared a coherent neryl acetate/γ-curcumene-rich EO profile, while showing descriptive variation in the relative abundance of several major and minor constituents. We observed marked differences among the six hydrosol headspace profiles analyzed under the same HS-SPME–GC–MS conditions. Eucalyptol showed the highest relative peak-area contribution in the four Capraia samples, whereas the Elba and Giglio profiles were characterized primarily by neryl acetate and oxygenated monoterpenes, respectively. The EO and hydrosol results provide complementary descriptive information; however, the relative percentages obtained using the two analytical workflows cannot be compared quantitatively. The differences observed among the analyzed pooled samples may reflect the combined influence of individual variability, local environmental conditions, sampling circumstances, genetic background, and distillation-related effects, which the present dataset cannot separate. Under the adopted DPPH assay conditions, the EO samples showed approximately 105- to 181-fold higher radical-scavenging capacity than the corresponding hydrosols. These preliminary in vitro results do not establish specific cosmetic or therapeutic efficacy, which would require dedicated biological, safety, stability, and sensory evaluations. Nevertheless, hydrosols represent potentially valuable co-products of essential-oil distillation, and their possible applications should be investigated through targeted biological and formulation studies. Because the study used a single pooled sample and a single distillation batch for each sampling site, without independent biological or distillation replication or absolute compound quantification, these patterns should be considered descriptive. The technical replicates included in the chemical analyses assess analytical repeatability but do not account for biological variability. Replicated collections, common-environment trials, and targeted chemical and biological validation are required before using the observed differences to support germplasm selection, product authentication, or population-specific quality standards.

## Figures and Tables

**Table 1:** Fresh aerial biomass processed by hydrodistillation, essential-oil volume recovered, and apparent extraction yield for six *H. italicum* subsp. *italicum* populations from the Tuscan Archipelago. Yield was calculated as mL of EO per 100 g of fresh biomass (% *v*/*w*).

Population	Fresh Biomass (g)	Essential Oil (mL)	Yield (% *v*/*w*, Fresh Weight)
CI	1606	2.20	0.14
CII	2183	3.00	0.14
CIII	2084	3.60	0.17
CIV	2150	4.00	0.19
E	2080	4.00	0.19
G	1800	2.90	0.16

**Table 2:** GC–MS profiles of the essential oils obtained from the six pooled site samples, expressed as relative peak-area abundance (%). Values are reported as mean ± standard deviation of three technical replicates. Technical replicates assess analytical repeatability and do not represent biological or distillation replication. nd, not detected; MH, monoterpene hydrocarbon; ME, monoterpene ester; OM, oxygenated monoterpene; SH, sesquiterpene hydrocarbon; OS, oxygenated sesquiterpene; AE, aliphatic ester; AD, aliphatic diketone. CI–CIV, Capraia samples; E, Elba; G, Giglio.

Compound	Class	CI	CII	CIII	CIV	E	G
neryl acetate	ME	30.02 ± 2.08	24.90 ± 2.00	29.5 ± 2.33	26.90 ± 1.98	29.17 ± 2.07	34.01 ± 2.09
γ-curcumene	SH	9.10 ± 0.98	12.19 ± 1.08	10.96 ± 0.99	11.71 ± 1.02	12.40 ± 1.22	14.91 ± 1.38
α-pinene	MH	8.63 ± 0.70	6.97 ± 0.72	7.70 ± 0.69	5.05 ± 0.66	14.17 ± 1.13	7.40 ± 0.98
neryl propionate	ME	9.08 ± 1.18	8.48 ± 0.88	9.14 ± 0.95	7.38 ± 0.89	4.37 ± 0.66	5.86 ± 0.70
limonene	MH	5.16 ± 0.61	4.84 ± 0.67	5.24 ± 0.70	6.69 ± 0.84	7.12 ± 0.80	9.71 ± 1.12
eucalyptol	OM	2.68 ± 0.08	7.98 ± 0.81	4.67 ± 0.33	6.10 ± 0.77	1.70 ± 0.09	1.01 ± 0.08
α-curcumene	SH	3.57 ± 0.33	4.08 ± 0.44	3.00 ± 0.30	4.23 ± 0.42	3.26 ± 0.39	2.74 ± 0.20
linalool	OM	2.17 ± 0.09	5.03 ± 0.35	4.18 ± 0.33	3.71 ± 0.23	3.20 ± 0.22	1.06 ± 0.10
rosifoliol	OS	1.72 ± 0.07	2.06 ± 0.12	3.01 ± 0.11	4.55 ± 0.23	3.21 ± 0.14	1.11 ± 0.09
4,6,9-trimethyldec-8-ene-3,5-dione	AD	1.07 ± 0.08	0.96 ± 0.07	nd	1.20 ± 0.09	2.15 ± 0.10	4.37 ± 0.22
italicene	SH	1.57 ± 0.07	1.97 ± 0.09	1.70 ± 0.08	1.55 ± 0.06	1.53 ± 0.09	1.72 ± 0.09
caryophyllene	SH	0.64 ± 0.02	1.94 ± 0.05	0.93 ± 0.03	1.19 ± 0.04	2.55 ± 0.10	1.73 ± 0.04
butyl angelate	AE	1.31 ± 0.04	2.43 ± 0.13	nd	2.32 ± 0.11	nd	nd
cis-α-bergamotene	SH	1.32 ± 0.07	1.63 ± 0.09	0.81 ± 0.02	1.40 ± 0.09	nd	0.58 ± 0.06
β-selinene	SH	1.21 ± 0.09	nd	nd	1.07 ± 0.08	2.18 ± 0.13	0.80 ± 0.03
4-terpineol	OM	1.37 ± 0.05	1.37 ± 0.06	nd	nd	0.72 ± 0.05	1.15 ± 0.09
α-terpineol	OM	0.58 ± 0.02	1.14 ± 0.09	1.10 ± 0.08	1.13 ± 0.10	0.57 ± 0.04	nd
β-pinene	MH	1.47 ± 0.07	nd	0.86 ± 0.06	nd	0.82 ± 0.01	1.20 ± 0.06
γ-terpinene	MH	1.20 ± 0.05	0.83 ± 0.01	0.75 ± 0.01	nd	0.46 ± 0.02	0.64 ± 0.04
α-selinene	SH	1.15 ± 0.06	nd	nd	0.92 ± 0.03	1.42 ± 0.09	nd
copaene	SH	2.41 ± 0.14	nd	nd	nd	nd	0.91 ± 0.04
fenchene	MH	nd	nd	0.87	nd	1.02 ± 0.78	1.35 ± 0.78
isobutyl angelate	AE	nd	1.27 ± 0.11	0.78 ± 0.07	1.05 ± 0.12	nd	nd
methylbutyl angelate	AE	nd	nd	2.11 ± 0.18	nd	nd	0.75 ± 0.05
4,6-dimethyloctane-3,5-dione	AD	nd	nd	1.54 ± 0.19	1.28 ± 0.09	nd	nd
trans-α-bergamotene	SH	0.79 ± 0.10	0.88 ± 0.11	nd	1.06 ± 0.09	nd	nd
geranyl angelate	ME	1.33 ± 0.10	1.37 ± 0.11	nd	nd	nd	nd
nerol	OM	nd	1.20 ± 0.09	0.64 ± 0.06	nd	nd	nd
3,5,7,10-tetramethylundec-9-ene-4,6-dione	AD	nd	nd	nd	nd	nd	1.37 ± 0.13
p-cymene	MH	1.08 ± 0.05	nd	nd	nd	nd	nd
longipinene	SH	nd	0.64 ± 0.03	nd	nd	nd	nd
ylangene	SH	nd	nd	nd	nd	nd	0.50 ± 0.05
β-farnesene	SH	0.34 ± 0.01	nd	nd	nd	nd	nd
Total monoterpenes		64.77 ± 5.08	62.74 ± 6.78	63.78 ± 0.65	56.96 ± 5.78	63.32 ± 5.58	63.39 ± 6.08
Total sesquiterpenes		23.82 ± 2.88	25.39 ± 2.66	21.49 ± 0.31	21.49 ± 0.27	26.55 ± 2.02	25.08 ± 1.99

**Table 3:** Volatile profiles of the hydrosols obtained from the six pooled site samples, determined by HS-SPME–GC–MS and expressed as relative peak-area abundance (%). Values are reported as mean ± standard deviation of three independent technical HS-SPME–GC–MS replicates. Technical replicates assess analytical repeatability and do not represent biological or distillation replication. nd, not detected; MH, monoterpene hydrocarbon; ME, monoterpene ester; OM, oxygenated monoterpene; SH, sesquiterpene hydrocarbon; OS, oxygenated sesquiterpene; AE, aliphatic ester; AD, aliphatic diketone. CI–CIV, Capraia samples; E, Elba; G, Giglio.

Compound	Class	Capraia I	Capraia II	Capraia III	Capraia IV	Elba	Giglio
eucalyptol	OM	94.56 ± 4.78	87.01 ± 4.08	46.94 ± 2.87	39.13 ± 3.08	nd	5.07 ± 0.38
artemisia ketone	OM	2.73 ± 0.35	nd	nd	nd	nd	nd
α-terpinolene	MH	1.00 ± 0.08	nd	nd	nd	nd	nd
camphor	OM	1.50 ± 0.14	nd	nd	nd	nd	nd
4-terpineol	OM	0.20 ± 0.06	2.45 ± 0.22	3.00 ± 0.48	3.97 ± 0.47	nd	16.56 ± 0.18
α-pinene	MH	nd	nd	nd	0.16 ± 0.04	nd	nd
camphene	MH	nd	nd	nd	0.05 ± 0.01	nd	nd
2,4-dimethylheptane-3,5-dione	AD	nd	nd	nd	0.43 ± 0.07	nd	nd
linalool	OM	nd	2.85 ± 0.12	6.95 ± 0.43	9.60 ± 0.15	nd	11.31 ± 0.84
pentyl 3-methylbut-2-enoate	AE	nd	nd	nd	0.79 ± 0.09	nd	nd
fenchyl alcohol	OM	nd	2.15 ± 0.18	4.21 ± 0.33	4.30 ± 0.30	nd	21.72 ± 1.96
nerol	OM	nd	1.31 ± 0.11	8.79 ± 0.73	4.79 ± 0.44	nd	29.81 ± 2.89
neryl acetate	ME	nd	4.23 ± 0.61	17.32 ± 0.20	18.99 ± 0.22	56.36 ± 0.69	1.05 ± 0.14
α-bergamotene	SH	nd	nd	nd	0.44 ± 0.08	nd	nd
7-isopropenyl-4,4,8,8-tetramethylbicyclo[4.2.0]octan-1,5-diol	OS	nd	nd	nd	1.89 ± 0.18	nd	nd
geranyl propionate	ME	nd	nd	8.01 ± 0.78	6.30 ± 0.62	11.50 ± 0.99	nd
bisabolol/cis-sesquisabinene hydrate	OS	nd	nd	nd	0.40 ± 0.04	nd	nd
α-curcumene	SH	nd	nd	4.78 ± 0.52	5.93 ± 0.65	20.59 ± 2.09	nd
α-selinene	SH	nd	nd	nd	0.56 ± 0.06	6.56 ± 0.70	nd
rosifoliol	OS	nd	nd	nd	2.26 ± 0.32	4.98 ± 0.53	6.23 ± 0.72
endo-borneol	OM	nd	nd	nd	nd	nd	1.98 ± 0.20
bisabolane	SH	nd	nd	nd	nd	nd	4.41 ± 0.60
α-eudesmol	OS	nd	nd	nd	nd	nd	0.79 ± 0.08
guaiol	OS	nd	nd	nd	nd	nd	1.07 ± 0.12

**Table 4:** DPPH radical-scavenging activity of the essential oils and hydrosols obtained from the six pooled site samples, expressed as micrograms of ascorbic acid equivalents per milliliter (µg AAE mL^−1^). Values are reported as mean ± standard deviation of four measurements obtained from two independent experiments; each performed in duplicate. CI–CIV, Capraia samples; E, Elba; G, Giglio; AAE, ascorbic acid equivalents.

Sample	Essential Oil (µg AAE mL^−1^)	Hydrosol (µg AAE mL^−1^)
Capraia I	325.60 ± 74.22	3.08 ± 0.69
Capraia II	448.66 ± 64.33	4.01 ± 0.58
Capraia III	454.99 ± 70.12	2.88 ± 0.41
Capraia IV	484.87 ± 67.45	2.68 ± 0.39
Elba	304.34 ± 63.11	1.95 ± 0.27
Giglio	289.99 ± 70.00	1.77 ± 0.32

**Table 5:** Geographic coordinates (WGS84) and altitude of the six sampling sites of *Helichrysum italicum* subsp. *italicum* collected in the Tuscan Archipelago.

Population	Latitude (N)	Longitude (E)	Altitude (m a.s.l.)
CI (Capraia)	43°02′57.9″	9°48′51.6″	350–400
CII (Capraia)	43°02′40.1″	9°50′49.2″	0–50
CIII (Capraia)	43°03′08.4″	9°50′14.4″	0–50
CIV (Capraia)	43°03′00.0″	9°49′44.5″	500–550
E (Elba)	42°43′28.4″	10°24′15.2″	350–400
G (Giglio)	42°21′14.4″	10°54′20.3″	350–400

## Data Availability

The original data presented in this study are included in the article. Further requests may be addressed to the corresponding author.
